# Results of Influenza Vaccination: Short Follow-Up Study of a Turkish Population

**DOI:** 10.1590/0037-8682-0605-2019

**Published:** 2020-09-11

**Authors:** Guzin Zeren Ozturk, Seda Ozmen, Memet Taskin Egici, Ekmel Burak Ozsenel

**Affiliations:** 1 Division of Family Medicine, Sisli Hamidiye Etfal Education and Research Hospital, Istanbul, Turkey.; 2 Division of Family Medicine, Haydarpasa Education and Research Hospital, Istanbul, Turkey.; 3 Division of Internal Medicine, Sisli Hamidiye Etfal Education and Research Hospital, Istanbul, Turkey.

**Keywords:** Upper respiratory disease, Influenza, Influenza vaccination, Turkey

## Abstract

**INTRODUCTION:**

The trivalent inactivated influenza vaccine was approved for use in Turkey during the 2018-2019 influenza season. We evaluated beliefs regarding the vaccine and vaccination outcomes in a Turkish population.

**METHODS:**

Individuals who were vaccinated with the trivalent inactivated influenza vaccine between November 1 and December 31, 2018, at the Sisli Hamidiye Training and Research Hospital in Istanbul, Turkey, were included in this study. A 15-item questionnaire was completed by a physician during a face-to-face interview with the participants. All participants were followed during the 2018-2019 influenza season through May 2019. The participants were instructed to consult the same physician in case of sudden illness. Participants’ beliefs and outcomes were assessed by their vaccination status for the 2017-2018 influenza season.

**RESULTS:**

A total of 150 participants were recruited. Their median age was 66 (range, 22-88) years. During the 2017-2018 influenza season, 4.1% had been hospitalized, 53.5% had developed an upper respiratory disease (URD), and 16.2% had been diagnosed with pneumonia. There were no cases of influenza, pneumonia, or hospitalization in the 2019 season; 49.3% of the participants developed a URD (n = 74). Among participants who had been vaccinated during both influenza seasons, 47.5% had had and/or developed a URD, with a higher number of cases during the 2018-2019 season.

**CONCLUSIONS:**

After vaccination, no cases of influenza, hospitalization, and pneumonia were observed and the incidence of URD decreased compared with that of the previous season.

## INTRODUCTION

Influenza is a viral respiratory infection that can lead to hospitalization or death. Some individuals, particularly the elderly, young children, and those with chronic diseases, are at a high risk of being infected^1^. Influenza is commonly observed in spring and winter every year and can cause pandemics. Globally, it is estimated that influenza results in 3-5 million cases of severe illness and 290,000-650,000 respiratory disease-related deaths each year^2^.

The optimal measure to prevent influenza is through vaccination with an inactivated virus. The Vaccines and Related Biological Products Advisory Committee of the U.S. Food and Drug Administration meets annually to select appropriate influenza viruses as components of the vaccine for the coming season^3^.

In Turkey, the trivalent inactivated influenza vaccine was approved for use during the 2018-2019 influenza season. According to the reports of the U.S. Centers for Disease Control and Prevention (CDC), the effectiveness of trivalent vaccine is between 70% and 90% in healthy adults^4^. A recent meta-analysis reported that the effectiveness of influenza vaccination was 59%,^5^ similar to the level of 50%-60% reported by the World Health Organization^6^. This difference of the effectiveness were because of the properties of the populations. In individuals of advanced age (>65 years), its effectiveness decreases as a result of increased frailty^7^. In Turkey, influenza vaccinations are promoted and funded by the state for individuals aged >65 years and for those in certain at-risk groups (diabetes, chronic lung disease, and heart diseases).

Vaccinations are performed in primary care settings from September to December. The protective effect of the vaccine begins 1-2 weeks after administration; in healthy adults it will remain effective for 6-8 months or longer^8^. The aim of the current study was to evaluate the protective effect of the trivalent inactivated influenza vaccine for vaccine recipients in Istanbul, Turkey. 

## METHODS

This prospective study was based at the Sisli Hamidiye Training and Research Hospital in Istanbul, Turkey. Eligible participants received the trivalent influenza vaccine between November 1 and December 31, 2018. The participants followed-up for 4 months until end of May 2019. All participants provided verbal and written permission and completed a 15-item questionnaire during a face-to-face interview with a physician. The questionnaire included basic sociodemographic features (age, sex, education, and income); the participant’s knowledge about influenza and vaccination; and their history of upper respiratory disease (URD), pneumonia, chronic diseases, or hospitalization during the previous (2018) influenza season. Sex was categorized male and female. Economic status was categorized into three levels according to the mean monthly income in Turkey for 2019 (national mean=2,200 Turkish liras). Educational status was categorized as less than a high school education, high school diploma, and college education or higher. 

Following national practice, participants who had been diagnosed with diabetes, chronic lung disease, and heart diseases were considered to be an at-risk group. All cases of influenza, URD, pneumonia, hospitalization occurring during the follow-up period were recorded (until end of May 2019). In cases of sudden illness, participants were instructed to consult the same physician. In addition, the physician called up every participant on the last day of each month to enquire about their health condition. When influenza was suspected, a rapid single-step diagnostic test using a nasal smear was performed (Humasis Influenza Antigen Card Plus, Humasis, Anyang, South Korea). 

### Ethical Approval

The study was approved by the Clinical Research Ethics Committee of Health Sciences University, Sisli Hamidiye Etfal Training and Research Hospital.

### Statistical Analysis

Descriptive statistics (number, percentage distribution, mean, and standard deviation) were calculated for all study variables. Abnormal data distributions as determined by the Shapiro-Wilk test (*P* < 0.001) were assessed using the Mann-Whitney U-test to compare groups. Logistic regression models were used to predict effect of sex, BMI, marital status, economic status, current smoking, current alcohol using, chronic diseases on URD (+). In the first stage, univariate regression analyses were performed for each variable; significant predictors were included in multivariate models. A model was created with classification tables from regression outputs. Sensitivity and specificity values are calculated. *P*-values of <0.05 were considered statistically significant. All analyses were performed with SPSS software, version 25.0 (IBM, Chiago, Illinois, USA). 

## RESULTS

Data for 150 participants were collected. Their median age was 66 (range, 22-88) years. Among all participants, 54.7% (n = 82) were women and 82.7% (n = 124) were married. Most of the participants had a low educational status (n = 80; 53.3%), a low economic status (n = 57; 38%), and were overweight (n = 65; 43.3%). Further, 18% of the participants were current smokers (n = 27) and 8.7% were current drinkers (n = 13). As seen in Table 1, whether there is any influenza risk group at participants’ home was questioned and the most were elderly (n = 75; 50%). Most of the at-risk participants had diabetes mellitus (n = 54; 36%).

As shown in Table 2, 136 (90.7%) participants were familiar with influenza vaccination, 99 (66%) had a history of influenza vaccination [most had been informed by a physician (n = 62; 41.3%)], and 58% (n = 87) believed that influenza is fatal. The primary reason for receiving an influenza vaccination was a physician’s recommendation (n = 78; 51.3%), while the most common reason for not having been previously vaccinated was a lack of knowledge (n = 18; 12%). 

**TABLE 1:** Distribution of sociodemographic features of participants.

	N	%
Gender		
Female	82	54.7
Male	68	45.3
Education Status		
Low (less than high school diploma)	24	16
Mid (high school diploma)	80	53.3
High (college or higher)	46	30.7
Economic Status		
Low (≤2200 TL)	57	38
Mid (2200-4400 TL)	49	32.7
High (≥4400 TL)	44	29.3
Marital Status		
Single	26	17.3
Married	124	82.7
Body Mass Index		
<25	58	38.7
25-29.9	65	43.3
≥30	27	18
Smoking		
Smoker	27	18
Non-smoker	123	82
Alcohol		
Drinker	13	8.7
Non-drinker	137	91.3
Chronic Diseases and Risk Groups***		
Chronic Cardiac Disorder		
Yes	43	29
No	107	71
Chronic Pulmonary Disorder		
Yes	37	25
No	113	75
Diabetes		
Yes		
No	54	36
	96	64
Renal Disease		
Yes	9	6
No	141	94
Immunosuppressive Conditions (HIV, Splenectomy, etc.)		
Yes	6	4
No	144	96
Specific Risk Groups Only (aged 65 and over, Health Professionals, etc.)		
Yes	43	29
No	107	71
Risk Groups at Participants’ Home***		
Young children	9	6
Pregnant women	6	4
Elder adult	75	50
Health professional	17	12
No risk group	48	33
Vaccination in Previous Season (2017-2018)		
Yes	51	34
No	99	66
Having Urd in Previous Season (2017-2018)		
Yes	89	59.3
No	61	40.7
Having Pneumonia in Previous Season (2017-2018)		
Yes	25	16.7
No	125	83.3
Hospitalized in Previous Season (2017-2018)		
Yes	9	6
No	141	94

*** Participants can have more than one condition.

**TABLE 2:** Participants’ responses about influenza and vaccine.

	N	%
**Belief that influenza was lethal**		
Yes	87	58
No	31	20.7
Unknown	32	21.3
**Knowledge about influenza**		
Yes	136	90.7
No	14	9.3
**Source of information**		
Media	37	24.7
Physician advice	62	41.3
Social circle	25	16.7
Allied health personnel	12	8
**Vaccinated in previous influenza season**		
Yes	99	66
No	51	34
**Reason vaccinated previously**		
Due to doctor’s proposal	77	51.3
Due to social circle’s proposal	8	5.3
Due to allied health personnel’s proposal	13	8.7
**Reason not vaccinated previously**		
Due to the lack of knowledge	18	35
Due to the thought of unnecessary	16	31
Due to not informed by doctor	15	30
Because of the opinion of ineffectiveness	2	4

The statistical relationship between the belief that influenza is lethal and history of influenza vaccination (*P* = 0.003) and educational status (*P* = 0.031) was significant. The relationship between the belief that influenza is fatal and familiarity with the influenza vaccination was not significant (*P* = 0.064) (Table 3). History of influenza vaccination was also significantly related to the increased educational status and increased economic status (*P* < 0,05). 

**TABLE 3:** Relationship between belief that influenza was lethal and education status, knowledge about influenza, vaccinated in previous season.

	Belief that influenza was lethal						p
	Yes		No		Unknown		
	N	%	N	%	N	%	
Education Status							**0.031**
Low (less than high school)	37	42.5	34	39.1	16	18.4	
Mid (high school diploma)	21	67.7	7	22.6	3	9.7	
High (college or higher)	22	68.8	5	15.6	5	15.6	
Knowledge About Influenza							0.064
Yes	83	61	26	19.1	27	19.9	
No	4	28.6	5	35.7	5	35.7	
Vaccinated in Previous Season							**0.003**
Yes	67	67.7	15	15.2	17	17.2	
No	20	39.2	16	31.4	15	29.4	

A total of 31 (20.7%) participants experienced side effects of the vaccination, with 17.3% (n = 26) reporting pain at the vaccination site. Other side effects included color and redness at the vaccination site (4.6% and 2.6%, respectively). The mean age of the group which had side effect after vaccination was lower than the group who didn’t any side effect (60.45 ± 18.8, 61.20 ± 16.1, respectively). Side effects were reported more often by women more than men (23.1%, 17.6%, respectively). The remaining sociodemographic factors (age, sex, economic status and educational status) were not significantly associated with the occurrence of side effects (P ≥ 0.05 for all comparisons).

During the previous (2017-2018) influenza season, 51 (34%) of the participants had received an influenza vaccination. Of all participants, 59.3% (n = 89) had developed an URD, 16.7% (n = 25) had been diagnosed with pneumonia, and 6% (n = 9) had been hospitalized (Table 1). The median of having URD was 1 (0;10); having pneumonia was 0 (0;4) and hospitalization was 0(0;2). When participants were compared by their 2017-2018 vaccination status, cases of URD (53.5%), pneumonia (16.2%), and hospitalization (4.1%) were lower in the vaccinated group.

During the 2019 influenza season, none of the participants developed influenza, pneumonia, or required hospitalization; the rate of URD was 49.3% (n = 74). Regarding sex, the prevalence of URD(+) was higher in women than in men (odds ratio (OR) = 2.51, *P* = 0.015). During both influenza seasons, 47.5% of the participants were vaccinated, with the vaccination rate being lower during the previous season than during the 2018-2019 season. 

Results of the logistic regression model predicting the development of a post-vaccination URD in the 2018-2019 season are shown in Table 4. Participant sex (*P* = 0.015) and economic status (*P* = 0.011) were found to be significant variables using the logistic model. Compared to the low income level, medium or higher incomes were associated with a significantly lower risk of URD(+) (OR = 0.25, *P* = 0.005; OR = 0.18, *P* = 0.012). BMI; marital status; smoking habit; drinking habit; as well as presence of diabetes, renal dysfunction, immune suppression, lung disease, and cardiac disease were not significantly associated with URD(+). 

**TABLE 4:** Results of multivariate logistic regression analysis of predictors of URD.

Variables	OR (95% CI)	p
**Gender**		
Male	1.00	
Female	2.51 (1.19-5.30)	**0.015**
**BMI**		
<25	1.00	
25.0-29.9	0.84 (0.35-2.01)	0.699
30>	1.78 (0.58-5.38)	0.307
**Marital Status**	0.62 (0.23-1.65)	0.340
**Economic Status**		
<2200 Turkish liras	1.00	
2200-4400 Turkish liras	0.25 (0.09-0.66)	**0.005**
>4400 Turkish liras	0.18 (0.04,0.69)	**0.012**
**Current Smoking** (yes)	1.17 (0.44-3.11)	0.741
**Current Alcohol Use** (yes)	1.21 (0.30-4.76)	0.783
**Diabetes Mellitus** (yes)	0.78 (0.35-1.72)	0.543
Renal Disease (yes)	0.66 (0.12-3.41)	0.623
Immunosuppressed (yes)	0.42 (0.06-2.62)	0.354
Chronic Pulmonary Disorder (yes)	0.79 (0.33-1.88)	0.607
Chronic Cardiac Disorder (yes)	0.80 (0.35-1.83)	0.602

The model accurately identified 53 of 76 participants without URD(−) (specificity = 69.7%) and 26 of 74 participants with URD(+) (sensitivity = 64.9%). Its overall estimation rate was 67.3%. Only 20% of the factors indicative of URD(+) were explained by the variables in the logistic model (R^2^ = 0.20; −2 log likelihood = 183.7).

The number of cases of URD after influenza vaccination was positively correlated to those reported for the previous influenza season (*r* = 0.24; *P* = 0.003), with URD commonly detected in those with renal disease.

When participants were asked about the effectiveness of influenza vaccination, 78% (n = 117) reported that it is effective, and 90% (n = 135) expressed the desire to be vaccinated during the next influenza season and that they would recommend the vaccine to their relatives.

## DISCUSSION

According to the CDC, influenza vaccination has been shown to reduce the frequency of physician visits by 40%-60%^9^. In our study, we did not observe any cases of influenza, pneumonia, or hospitalization after influenza vaccination. This may be because of the similarity between influenza and other circulating viruses.

Influenza vaccination also reportedly reduces the prevalence of influenza-like illnesses, lost days at work and school, and physician’s visits^10^ ^,^ ^11^. In our study, the prevalence of URD was 43.1% in the vaccinated groups during both influenza seasons and was lower than the overall rates for both the previous (2017-2018) and current (2018-2019) seasons. This suggests that influenza vaccination reduces the prevalence of URD. A decreased prevalence of URD was also found in a previous study conducted in Turkey^12^. Influenza vaccination stimulates the immune system, increasing protection against other viruses because of the cross-sectional protection of memory CT8 T cells against different influenza A subtypes^13^ ^,^ ^14^. The prevalence of URDs was related to advanced age and male sex. Some studies have reported a decline in the effectiveness of influenza vaccination in older adults^15^ from impaired cell-mediated immunity^16^. Otherwise, a low economic status and presence of renal disease are the most frequent risk factors for URD. Many studies have shown a negative relationship between the glomerular filtration rate and risk of death from infection^17^ ^,^ ^18^. This is associated with a change in the primary host defense mechanisms in renal disease^19^.

Vaccination reduces the rate of infection and is considered a human right similar to clean water^20^. However, myths associated with vaccination are fairly common in Turkey, such as a belief that vaccination is only for children, and vaccination rates are generally low in adults (44.8% for >19 years)^15^. Remarkably, 40,000-80,000 adults die from infection and a considerable number of adults are hospitalized for vaccine-preventable diseases such as influenza, pneumococcal disease, zoster, and pertussis. According to a study in 2010, this resulted in a cost of $15 billion^21^. National rates for Turkey have not been reported but local studies report averages of 35%^22^ ^,^ ^23^. 

Influenza in Turkey results in a pandemic every year, with different names being used on social media and in the news. According to some studies, influenza vaccine is the most commonly known vaccine among adults^24^. In our study, the rate of familiarity with influenza vaccine was high (90.7%). However, the rate of the participants who believed that influenza is lethal was lower than the rate of vaccination (58%). We found relationships between the belief that influenza is lethal, a history of influenza vaccination, and educational status. However, these factors were not related to answers about familiarity with the influenza vaccine. This may be due to a confusion between the common cold and influenza among many individuals, and possibly because of a myth that vaccination actually causes influenza and is unsafe^25^. 

In studies conducted on barriers to adult vaccination, the most common reason was typically that patients had not been informed by their physician^26^ ^,^ ^27^. Similarly, in our study the most common reason for vaccination was physicians’ recommendation (n = 78; 51.3%). Vaccination rates also increased with higher economic status and educational status^28^. The most common reason for not being vaccinated was a lack of knowledge (n = 18; 12%), highlighting the importance of public information and physicians’ opinions about vaccination.

There are two types of side effects to vaccination-local and systemic. Although systemic side effects were not observed in our study, pain at the vaccination site was the most frequent local side effect (n = 26; 17.3%) which is similar to previous findings^29^ ^,^ ^30^. Although there were no significant differences, the prevalence of side effects was higher among women and younger participants, which is also similar to previous findings^31^ ^,^ ^32^. Thus, current trivalent influenza vaccine appears to be well-tolerated^33^ ^,^ ^34^.

## CONCLUSIONS

After influenza vaccination, our study did not detect any cases of influenza, hospitalization, or pneumonia. In addition, the prevalence of URD decreased during the 2018-2019 influenza season compared to the previous season. No systemic side effects were reported, and the influenza vaccination was well-tolerated. However, vaccination rates remain very low mainly because of insufficient knowledge regarding the risk of influenza infections, and the protection afforded by vaccines, among physicians as well as the general public. In primary care settings, promotion of accurate information about vaccines and influenza by physicians is likely to increase vaccination rates.
